# Beyond disease counts: multimorbidity patterns and self-rated health in South African adults–a network analysis of the 2016 demographic and health survey

**DOI:** 10.3389/fpubh.2026.1947329

**Published:** 2026-10-20

**Authors:** Olalekan A. Uthman, Matthew Hazell, Muhammed Mubashir Babatunde Uthman, Kolawole W. Wahab, Andre Pascal Kengne, Paramjit Gill, Ponnusamy Saravanan

**Affiliations:** 1 Warwick Applied Health, Warwick Center for Global Health, Warwick Medical School, University of Warwick, Coventry, United Kingdom; 2 Warwick Applied Health, Warwick Medical School, University of Warwick, Coventry, United Kingdom; 3 Department of Epidemiology and Community Health, College of Health Sciences, University of Ilorin and University of Ilorin Teaching Hospital, Ilorin, Nigeria; 4 Department of Medicine, University of Ilorin, Ilorin, Nigeria; 5 Non-Communicable Diseases Research Unit, South African Medical Research Council, Cape Town, South Africa; 6 African Population and Health Research Center, Nairobi, Kenya; 7 Department of Diabetes, Endocrinology and Metabolism, George Eliot Hospital, Nuneaton, United Kingdom

**Keywords:** disease clustering, HIV, multimorbidity, network analysis, non-communicable diseases, self-rated health, South Africa

## Abstract

**Background:**

Multimorbidity is rising rapidly in sub-Saharan Africa, where infectious and non-communicable disease burdens overlap. Whether the pattern of co-occurring conditions carries information beyond a simple disease count is unclear.

**Objective:**

To map patterns of co-occurring chronic conditions in South African adults, test for additive interactions between condition pairs, and compare network-derived measures with the disease count as correlates of poor self-rated health.

**Methods:**

Cross-sectional analysis of the nationally representative 2016 South Africa Demographic and Health Survey. The analytic sample comprised 5,650 adults aged 18 years and older with complete data on 12 chronic conditions ascertained by self-report and biomarker measurement. The outcome was self-rated health, dichotomised as poor vs. average, good or excellent. Disease networks were constructed from phi coefficients with Louvain community detection; cluster stability used 1,000 design-consistent bootstrap replicates. Survey-weighted logistic regression adjusted for age, sex and residence compared the disease count with network-based measures (per 1-SD increase), using the area under the receiver operating characteristic curve (AUC), DeLong tests and cross-validation.

**Results:**

Three disease clusters emerged: infectious (tuberculosis, HIV, anemia), cardiometabolic and pain (hypertension, diabetes, chronic pain), and cardiovascular-respiratory-neoplastic (stroke, heart attack, high cholesterol, chronic bronchitis, asthma, cancer). The infectious cluster co-clustered in 100% of bootstrap replicates, and network structure differed by sex (permutation *p* = 0.001) and residence (*p* = 0.031). Adjusted odds ratios for poor self-rated health were 1.66 (95% CI 1.41–1.95) per SD of disease count, 1.55 (1.31–1.82) per SD of network score and 1.24 (1.03–1.49) per SD of max centrality; the network measures were highly correlated with disease count (*r* = 0.96) and did not improve discrimination (AUC 0.756 vs. 0.758; DeLong *p* = 0.26), a finding confirmed by cross-validation. One additive interaction remained after false-discovery-rate correction: stroke with anemia (+0.55 on the risk-difference scale, 95% CI 0.24–0.86).

**Conclusions:**

Network analysis revealed robust disease clustering, most notably a stable infectious cluster, and subgroup-specific patterns relevant to integrated care planning. Individual-level network measures did not outperform a simple disease count. Disease counts remain a pragmatic risk marker; pattern-based measures merit prospective evaluation before clinical adoption.

## 1 Introduction

Multimorbidity, the co-occurrence of multiple chronic conditions, is rising rapidly in low- and middle-income countries (LMICs) due to demographic and epidemiological transitions (1). Estimates of multimorbidity in South Africa vary widely with setting and age, from 3% to 23% in studies of younger or mixed-age populations and 30% to 87% among older adults, while a primary care morbidity survey reported multimorbidity in 14.4% of patients with non-communicable diseases (2, 3), with established associations to functional decline, disability, poor quality of life, and mortality (4, 5). However, the impact of multimorbidity on health extends beyond simply counting diseases. Specific disease combinations may have greater-than-additive effects on health outcomes (6). Traditional disease-counting approaches fail to capture these complex interactions (7). Network analysis maps relationships between multiple conditions simultaneously (8) to identify disease clusters, influential conditions, and additive interactions (9).

Rzewuska et al., Wang et al. (10, 11) Most multimorbidity research in sub-Saharan Africa has described disease burden and groupings using prevalence counts or factor- and cluster-analytic approaches (2, 3, 10); studies that model the co-occurrence structure itself with network methods, representing conditions as nodes and their pairwise associations as weighted edges, remain rare (10, 11). South Africa is home to the largest number of people living with HIV in the world and is classified by the World Health Organization among high-burden countries for tuberculosis and HIV-associated tuberculosis (12, 13), alongside a rising burden of hypertension and diabetes. The overlap of these burdens may produce multimorbidity patterns unlike those described in high-income settings. Although South Africa is an upper-middle-income country, its public health system serves most of the population with constrained per-capita resources, making integrated approaches to multimorbidity particularly relevant.

This study applied network analysis to map multimorbidity patterns and their association with self-rated health among South African adults. We addressed three prespecified questions: (i) which conditions cluster together, and whether the clusters are stable under resampling; (ii) whether specific condition pairs show greater-than-additive associations with poor self-rated health; and (iii) whether individual-level network-based measures of multimorbidity improve on a simple disease count as correlates of poor self-rated health. We hypothesized distinct infectious and cardiometabolic clusters, subgroup differences by sex and place of residence, and incremental value of network-based measures over disease count.

## 2 Materials and methods

### 2.1 Study design and population

We analyzed cross-sectional data from the 2016 South Africa Demographic and Health Survey (SADHS), a nationally representative household survey using stratified two-stage probability sampling; the adult health module, including biomarker measurement, was administered to one randomly selected adult per household. Of 10,336 adult health module respondents, 868 aged under 18 years were excluded, leaving 9,468 adults; a further 3,818 were excluded because at least one of the 12 study conditions could not be ascertained, almost entirely reflecting the biomarker subsample design (HIV serostatus unavailable for 3,485; glycated hemoglobin for 3,303; hemoglobin for 2,880; blood pressure for 2,187). The analytic sample therefore comprised 5,650 adults with complete data on all 12 conditions (complete-case analysis; per-variable missingness is reported in Supplementary Table S2). The SADHS 2016 protocol was approved by the South African Medical Research Council Ethics Committee and the ICF Institutional Review Board, and all participants provided written informed consent. This secondary analysis used publicly available de-identified data and did not require further ethical approval. The study is reported in accordance with the STROBE statement for cross-sectional studies (completed checklist in the Supplementary Material).

### 2.2 Outcome and exposures

The primary outcome was self-rated health, measured with the single SADHS item “In general, how would you describe your health?” with response options excellent, good, average and poor. The outcome was dichotomised as poor vs. average, good or excellent, isolating the clearly negative end of the scale; the full four-category distribution is reported in Table 1, and sensitivity analyses examined the alternative dichotomisation (poor or average vs. good or excellent) and every cumulative cut of the scale (Supplementary Table S10). We assessed 12 chronic conditions through self-report and/or biomarker measurement: tuberculosis, hypertension (measured blood pressure ≥140/90 mmHg or current treatment), stroke, high cholesterol, anemia (hemoglobin < 12 g/dL for women, < 13 g/dL for men, using DHS altitude-adjusted values), chronic bronchitis, diabetes (glycated hemoglobin ≥6.5% or current treatment), asthma, cancer, heart attack, HIV (laboratory-confirmed serostatus), and chronic pain (persistent >3 months). Self-reported conditions refer to ever having been told by a health professional that the respondent had the condition. The exact SADHS variables and operational definitions for every condition are given in Supplementary Table S1. Sociodemographic covariates included age, sex, and urban vs. rural residence.

**Table 1:** Characteristics of the 5,650 South African adults included in the analysis, 2016 South Africa demographic and health survey.

Characteristic	Category/statistic	*n*	Weighted estimate	SE	95% CI
**Sociodemographic profile**					
Age (years)	Mean (SE)	5,650	32.7	0.23	32.3 to 33.2
	Median (IQR)	5,650	31.0 (24.0 to 41.0)	—	—
Self-rated health status	Poor	747	7.7%	0.52	6.7 to 8.8
	Average	1,926	28.9%	0.97	27.1 to 30.9
	Good	2,326	47.6%	1.05	45.5 to 49.7
	Excellent	651	15.8%	0.89	14.1 to 17.6
Sex	Male	2,100	43.0%	1.01	41.0 to 45.0
	Female	3,550	57.0%	1.01	55.0 to 59.0
Place of residence	Urban	2,886	63.2%	1.69	59.9 to 66.5
	Rural	2,764	36.8%	1.69	33.5 to 40.1
Ethnicity	Black/African	4,955	88.9%	1.19	86.4 to 91.1
	Mixed/Colored	432	6.6%	0.76	5.2 to 8.2
	White	213	3.4%	0.84	2.1 to 5.5
	Asian/Other	50	1.1%	0.36	0.6 to 2.1
**Chronic disease burden**					
Disease count	Mean (SE)	5,650	1.22	0.026	1.17 to 1.28
	Median (IQR)	5,650	1.00 (0.00 to 2.00)	—	—
Disease count distribution	No conditions	1,257	29.5%	0.99	27.5 to 31.4
	1 condition	1,820	36.0%	0.98	34.1 to 37.9
	2 conditions	1,480	22.8%	0.81	21.3 to 24.5
	3 conditions	685	7.7%	0.54	6.7 to 8.8
	4 conditions	295	3.0%	0.38	2.3 to 3.8
	≥5 conditions	113	1.1%	0.22	0.7 to 1.6
**Individual chronic conditions: cardiometabolic**					
Hypertension	Yes	2,724	34.2%	1.20	31.9 to 36.6
Diabetes	Yes	1,336	15.0%	0.72	13.7 to 16.5
High blood cholesterol	Yes	182	1.9%	0.30	1.3 to 2.6
**Individual chronic conditions: cardiovascular**					
Heart attack	Yes	247	2.2%	0.34	1.6 to 3.0
Stroke	Yes	84	0.8%	0.15	0.6 to 1.1
**Individual chronic conditions: infectious**					
HIV	Yes	1,169	24.0%	1.01	22.1 to 26.0
Tuberculosis	Yes	92	1.7%	0.24	1.3 to 2.2
**Individual chronic conditions: respiratory and pain**					
Chronic pain	Yes	1,239	16.6%	1.05	14.7 to 18.8
Asthma	Yes	219	2.8%	0.33	2.2 to 3.5
Chronic bronchitis	Yes	79	0.9%	0.19	0.6 to 1.3
**Individual chronic conditions: other**					
Anemia	Yes	1,196	21.7%	1.10	19.7 to 24.0
Cancer	Yes	61	0.7%	0.15	0.4 to 1.0
**Network-based measures**					
Network score	Mean (SE)	5,650	0.31	0.007	0.29 to 0.32
	Median (IQR)	5,650	0.30 (0.00 to 0.38)	—	—
Max centrality	Mean (SE)	5,650	0.56	0.009	0.54 to 0.57
	Median (IQR)	5,650	0.82 (0.00 to 0.82)	—	—
Max centrality distribution	No conditions (0.00)	1,257	29.5%	0.99	27.5 to 31.4
	Low (0.18)	259	6.2%	0.55	5.2 to 7.3
	Moderate (0.73)	23	0.5%	0.13	0.3 to 0.8
	High (0.82)	2,611	44.1%	1.26	41.6 to 46.6
	Very high (0.91)	1,500	19.7%	1.06	17.7 to 21.9

*n*, unweighted number of participants; SE, standard error; CI, confidence interval; IQR, interquartile range.

All estimates are survey-weighted and incorporate the SADHS sampling weights, strata and primary sampling units, with standard errors obtained by Taylor linearisation. Unweighted counts are reported alongside weighted estimates because the adult health module was administered to one randomly selected adult per household, so unweighted percentages do not describe the national population.

Confidence intervals for percentages are logit-transformed, which keeps the interval within the 0 to 100% range and is preferable for the rarer conditions.

Medians are shown with the interquartile range as a measure of spread; percentile-based confidence limits are not reported because the disease count and the derived network measures take few distinct values.

The network score is the sum of the eigenvector centralities of the conditions an individual has. Max centrality is the highest degree centrality among an individual's conditions, ranging from 0 (no chronic conditions) to 0.91 (presence of a maximally connected hub condition).

### 2.3 Statistical analysis

#### 2.3.1 Construction of the multimorbidity network

We constructed a network where each of the 12 chronic conditions was a node, and connections between nodes were weighted by how often the two conditions occurred together. For each pair of conditions we calculated the phi coefficient, the correlation between two binary variables. Phi is bounded by the prevalence of each condition, so pairs involving rare conditions cannot attain high values even when they co-occur strongly; we therefore repeated the analysis using observed-to-expected co-occurrence ratios as a sensitivity check. Stronger associations (indicating conditions that frequently appear together) received heavier weights. Edges were retained where the phi coefficient was positive (phi > 0); no additional magnitude threshold was applied, so 52 of the 66 possible edges were retained, and the 14 condition pairs with phi ≤ 0 (predominantly pairs involving HIV) carried no edge (Supplementary Table S3). These edge rules were operational analytic choices rather than formally prespecified criteria; the sensitivity analyses described below assess their influence. Network analyses were performed in Python (version 3.11) (NetworkX); design-based regression models used the R survey package (version 4.5), and co-occurrence statistics and resampling procedures used Python (SciPy, statsmodels, scikit-learn) and R.

#### 2.3.2 Identifying disease clusters

To identify groups of conditions that cluster together, we applied the Louvain community detection algorithm (NetworkX implementation, resolution parameter 1.0, fixed random seed), which groups diseases that occur together more frequently within clusters than between clusters (14). We assessed the reliability of these clusters with 1,000 bootstrap replicates in which primary sampling units were resampled with replacement within strata, respecting the survey design; in each replicate the network and communities were re-estimated, and we report the label-invariant proportion of replicates in which each pair of conditions was assigned to the same cluster (Supplementary Table S4).

#### 2.3.3 Measuring condition importance: centrality at the population level

We calculated three measures of how ‘important' or ‘influential' each condition is within the overall network: ***Degree centrality*** measures how many other conditions a given disease tends to co-occur with. A condition with high degree centrality frequently appears alongside many different diseases. ***Betweenness centrality*** identifies conditions that bridge otherwise separate disease groups. A condition with high betweenness sits at the intersection of multiple disease clusters and may represent shared biological pathways or risk factors. ***Eigenvector centrality*** accounts for both the number of connections a condition has and how well-connected those neighboring conditions are. A disease with high eigenvector centrality not only co-occurs with many conditions but specifically with conditions that are themselves highly interconnected, placing it at the core of the multimorbidity network rather than at the periphery (15). All centrality measures were computed on the thresholded network retaining only positive-phi edges.

#### 2.3.4 Measuring multimorbidity complexity: network measures at the individual level

Beyond simply counting conditions, we derived two network-based measures to capture the complexity and interconnectedness of each individual's multimorbidity 'profile. First, the network score quantified how strongly an individual's conditions aligned with the broader population disease network, calculated as the sum of eigenvector centrality values for all conditions present; higher scores indicate multimorbidity patterns characterized by common, mutually connected conditions rather than isolated or atypical combinations. Second, max centrality captured whether an individual had at least one highly connected “hub” condition, defined as the highest degree centrality among their conditions; higher values suggest the presence of a sentinel disease that is frequently linked to complex multimorbidity even when co-occurring conditions are less interconnected. We used disease count (the total number of chronic conditions) as the primary comparator, reflecting its widespread use and ease of interpretation despite treating conditions as equivalent and ignoring disease-specific interactions (16). Because the network score is a sum over the conditions an individual has, it is intrinsically correlated with the disease count; we therefore report correlations and variance inflation factors between disease count and both network-based measures, and we compare these predictors using prespecified nested models and validated discrimination rather than coefficient magnitudes.

#### 2.3.5 Testing for additive interactions

We tested whether condition pairs were associated with poor self-rated health beyond the sum of their individual associations (greater-than-additive interaction on the risk-difference scale). For each condition pair with at least 20 participants having both conditions (47 of the 66 possible pairs; the remaining 19 sparse pairs were suppressed as prespecified), we fitted a survey-weighted linear-probability model containing both conditions and their product term and estimated the interaction contrast with design-based 95% confidence intervals. We controlled the false discovery rate across all tested pairs using the Benjamini–Hochberg procedure and report joint counts, interaction contrasts, confidence intervals and adjusted q-values for every pair (Supplementary Table S8). We use the term additive interaction descriptively; no biological synergy is implied.

#### 2.3.6 Predictive models of self-reported health

We compared the disease count with the network-based measures as correlates of poor self-rated health using logistic regression models that incorporated the survey sampling weights, strata and primary sampling units (Taylor linearisation; R survey package) and adjusted for age, sex, and urban vs. rural residence. Continuous predictors (disease count, network score, max centrality) were standardized, and their odds ratios are expressed per 1-SD increase; disease count is additionally reported per additional condition. We compared prespecified nested models–covariates only; covariates plus disease count; plus network score; plus max centrality; plus disease count and both network measures; and plus the 12 conditions entered individually–on discrimination (area under the receiver operating characteristic curve, with paired DeLong tests) and on the design-based Akaike information criterion. To address optimism from deriving and evaluating the network measures in the same sample, we additionally performed 10-fold stratified cross-validation in which the network, centralities and individual-level scores were recomputed within each training fold before evaluation on the held-out fold. We report odds ratios with 95% confidence intervals.

All prevalence estimates, logistic regression models and interaction models incorporated the SADHS sampling weights, strata and primary sampling units. The primary co-occurrence network was estimated unweighted, because phi coefficients describe the conditional co-occurrence structure of the analytic sample; a fully survey-weighted network was estimated as a sensitivity analysis (Supplementary Table S6), and bootstrap resampling respected the design by resampling primary sampling units within strata. Label-permutation tests for subgroup comparisons were performed on unweighted networks.

#### 2.3.7 Subgroup analyses

To examine whether multimorbidity patterns vary across population subgroups, we constructed separate disease networks stratified by sex (male vs. female), and place of residence (urban vs. rural). Within each subgroup, we repeated the clustering analysis, calculated centrality measures, and estimated additive interactions. To test whether networks genuinely differed between subgroups, we permuted group labels 1,000 times, rebuilt both networks at each iteration, and compared the observed summed absolute difference in edge weights with the resulting permutation distribution, which represents the differences expected if both groups shared a single underlying network. Permutation *p*-values are reported for each comparison.

## 3 Results

### 3.1 Sample characteristics

Table 1 reports unweighted counts with survey-weighted percentages. The weighted sample was 57.0% female (unweighted 62.8%, reflecting the module design), with a weighted mean age of 32.7 years (median 31, IQR 24–41); 36.8% lived in rural areas. The sample reflected a dual disease burden, with high weighted prevalences of both infectious conditions (HIV 24.0%, tuberculosis 1.7%) and non-communicable conditions (hypertension 34.2%, diabetes 15.0%, anemia 21.7%, chronic pain 16.6%). Overall, 70.5% of participants (weighted) had at least one chronic condition and 34.6% had two or more. Weighted, 7.7% of adults rated their health as poor (unweighted 747/5,650, 13.2%).

### 3.2 Disease clustering patterns

#### 3.2.1 Overall disease clustering patterns

Network analysis of the full sample identified three distinct clusters of co-occurring chronic conditions (Figure 1A). Cluster 1 comprised three conditions: tuberculosis, anemia, and HIV. These conditions formed a tightly interconnected group separate from other diseases. Cluster 2 was the largest and included six conditions: stroke, heart attack, high blood cholesterol, chronic bronchitis, asthma, and cancer. This cluster encompassed cardiovascular, respiratory, and neoplastic conditions. Cluster 3 consisted of three conditions: hypertension, diabetes, and chronic pain. These conditions formed a distinct group linking metabolic diseases with chronic pain. The three-cluster solution was reproducible under resampling: pairs assigned to the same cluster in the main analysis co-clustered in a mean of 89% of 1,000 design-consistent bootstrap replicates (minimum 75%), compared with 21% for cross-cluster pairs, and the tuberculosis-HIV-anemia and hypertension-diabetes pairings co-clustered in 100% of replicates (Supplementary Table S4). The infectious cluster and the eigenvector centrality ranking were unchanged in sensitivity networks based on observed-to-expected co-occurrence ratios and on survey-weighted phi coefficients, although the boundary between the two non-communicable clusters varied (Supplementary Tables S5, S6).

**Figure 1:**
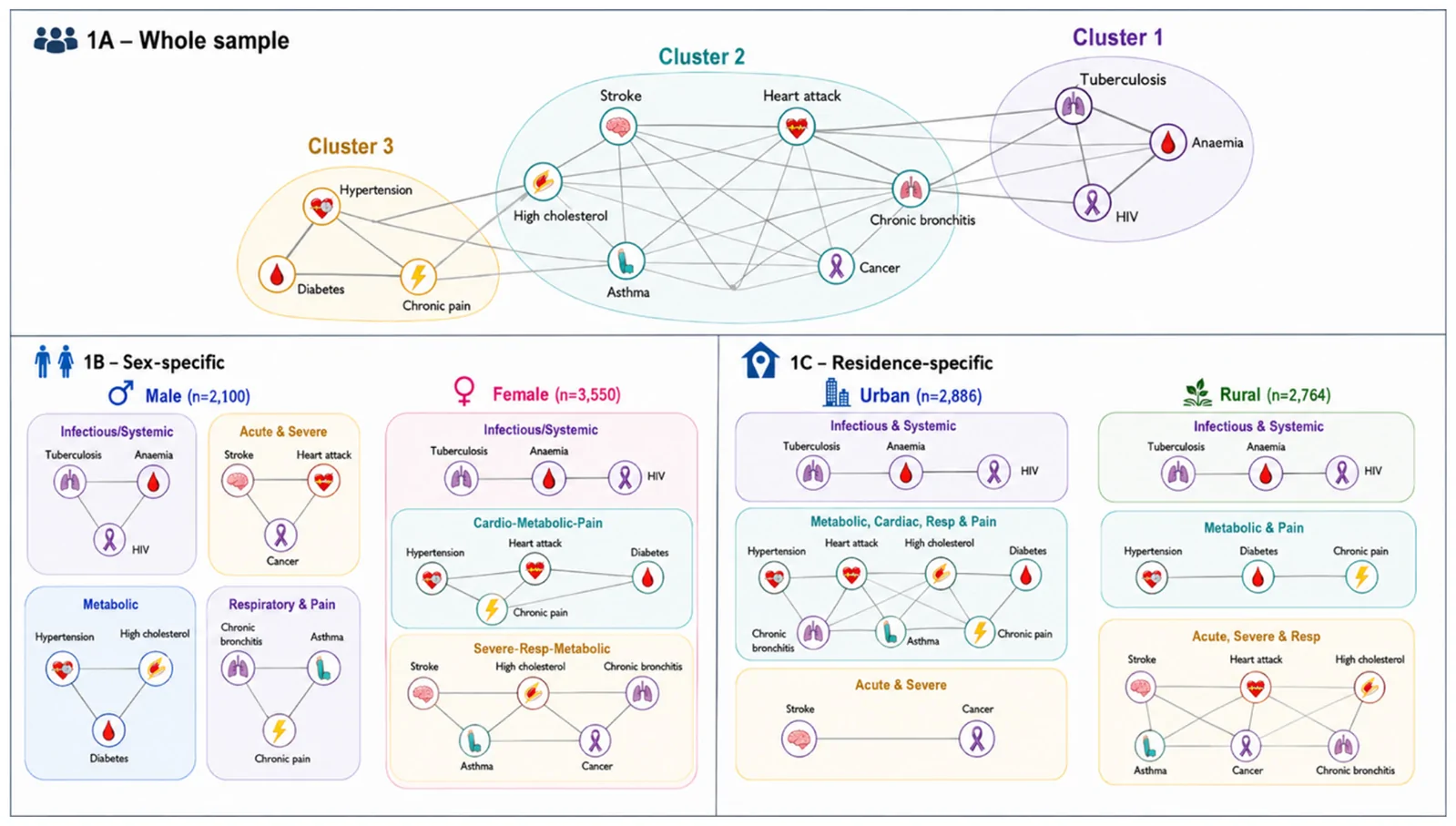
Multimorbidity disease networks in South African adults, overall and by sex and place of residence. **(A**) Overall multimorbidity network. Network diagram showing co-occurrence patterns among 12 chronic conditions in 5,650 South African adults. Each circle (node) represents one chronic condition. Lines (edges) connect conditions that frequently co-occur, with line thickness proportional to the phi coefficient; edges are shown where the phi coefficient was positive (phi > 0). Colors indicate disease clusters identified by community detection analysis: Cluster 1 (purple) = infectious and hematological diseases (tuberculosis, HIV, anemia); Cluster 2 (teal/blue) = cardiovascular, respiratory, and neoplastic diseases (stroke, heart attack, high blood cholesterol, chronic bronchitis, asthma, cancer); Cluster 3 (yellow) = cardiometabolic diseases and pain (hypertension, diabetes, chronic pain). **(B)** Sex-stratified multimorbidity networks. Left: males (*n* = 2,100). Right: females (*n* = 3,550). While the infectious disease cluster (tuberculosis, HIV, anemia) remains stable across both sexes, males show distinct separation of conditions by disease category (four separate clusters), whereas females show greater integration across disease categories with cardiovascular events clustering alongside metabolic conditions and chronic pain (three clusters with cross-system integration). **(C)** Place of residence-stratified multimorbidity networks. Left: urban residents (*n* = 2,886). Right: rural residents (*n* = 2,764). The infectious disease cluster remains stable across both settings. Urban residents show broad integration of metabolic, cardiac, and respiratory conditions into a single large cluster, while rural residents show respiratory conditions (chronic bronchitis, asthma) clustering separately with acute severe events (stroke, heart attack, cancer) rather than with metabolic diseases.

#### 3.2.2 Sex-specific clustering patterns

Multimorbidity patterns differed markedly between males and females (Figure 1B). One cluster remained stable across both sexes: tuberculosis, anemia, and HIV consistently formed a distinct infectious disease cluster in males and females. Beyond this stable infectious cluster, males and females showed different disease grouping patterns. In males, conditions separated into four distinct clusters organized by disease category. Respiratory conditions (chronic bronchitis and asthma) clustered with chronic pain. Metabolic conditions (hypertension, high blood cholesterol, and diabetes) formed a separate cluster. Acute and severe conditions (stroke, heart attack, and cancer) comprised a third cluster. The infectious diseases (tuberculosis, anemia, HIV) formed the fourth cluster. In females, conditions showed greater integration across disease categories. Heart attack did not cluster with other acute cardiovascular events (stroke) as it did in males. Instead, heart attack grouped with metabolic conditions (hypertension and diabetes) and chronic pain in a single large cluster. Respiratory conditions (chronic bronchitis and asthma) clustered with stroke and high blood cholesterol rather than forming a separate respiratory group. The infectious disease cluster (tuberculosis, anemia, HIV) remained distinct, as in males. Permutation testing supported a genuine overall difference between male and female networks (summed absolute edge-weight difference 2.67; *p* = 0.001).

#### 3.2.3 Urban-rural clustering patterns

Disease clustering patterns also varied by place of residence (Figure 1C). As with sex differences, the tuberculosis-anemia-HIV cluster remained stable in both urban and rural settings. In urban areas, conditions showed broad integration. One large cluster combined metabolic conditions (hypertension, diabetes, high blood cholesterol), cardiac events (heart attack), respiratory diseases (chronic bronchitis and asthma), and chronic pain. Only stroke and cancer formed a separate small cluster distinct from this integrated group. In rural areas, conditions separated into more distinct groups. Hypertension, diabetes, and chronic pain formed one cluster. Respiratory conditions (chronic bronchitis and asthma) did not cluster with metabolic diseases as in urban areas. Instead, these respiratory conditions grouped with stroke, heart attack, high blood cholesterol, and cancer in a second large cluster. The infectious disease cluster (tuberculosis, anemia, HIV) remained separate, as in urban settings. Permutation testing also supported the urban-rural difference (summed absolute edge-weight difference 2.19; *p* = 0.031). Because the partition of the non-communicable conditions was less stable than the infectious cluster under resampling, these subgroup contrasts are best interpreted as differences in overall co-occurrence structure rather than in exact cluster memberships.

### 3.3 Identifying influential conditions within the network

Centrality measures identified which conditions were most interconnected within the multimorbidity network (Table 2). Degree centrality measures how many other conditions a given disease tends to co-occur with. Four conditions had the highest degree centrality scores (0.91): heart attack, chronic bronchitis, asthma, and chronic pain. Each of these conditions co-occurred with 10 of the 11 other chronic conditions. At the opposite end, HIV had the lowest degree centrality (0.18), co-occurring with only 2 of the 11 other conditions. Betweenness centrality identifies conditions that bridge different disease clusters. Anemia had the highest betweenness centrality (0.10), meaning it most frequently sat between other conditions in the network. HIV had zero betweenness centrality, indicating it did not bridge different disease groups. Eigenvector centrality measures whether a condition is connected to other highly connected conditions. Four conditions shared the highest eigenvector centrality scores (0.32): chronic bronchitis, asthma, heart attack, and chronic pain. These conditions were connected not only to many other diseases but specifically to diseases that were themselves highly interconnected. HIV had the lowest eigenvector centrality (0.06).

**Table 2:** Centrality measures of the chronic conditions in the multimorbidity network.

Chronic condition	Degree	Betweenness	Eigenvector
Tuberculosis	0.727	0.077	0.241
Hypertension	0.818	0.008	0.297
Stroke	0.818	0.005	0.300
Heart attack	0.909	0.012	0.324
Anemia	0.818	0.099	0.271
High blood cholesterol	0.818	0.008	0.297
Diabetes	0.818	0.005	0.300
Chronic bronchitis	0.909	0.012	0.324
Asthma	0.909	0.012	0.324
Cancer	0.818	0.005	0.300
HIV positive	0.182	0.000	0.056
Chronic pain	0.909	0.012	0.324

Centrality measures quantifying the importance and influence of each chronic condition within the overall multimorbidity network. Degree centrality measures how many other conditions a given disease tends to co-occur with (range: 0–1, with higher values indicating more connections; a value of 0.91 indicates the condition co-occurred with 10 of the 11 other conditions). Betweenness centrality measures how often a condition bridges different disease clusters (range: 0–1, with higher values indicating the condition sits between other conditions in the network). Eigenvector centrality measures whether a condition is connected to other highly connected conditions (range: 0–1, with higher values indicating the condition is connected not only to many other diseases but specifically to diseases that are themselves highly interconnected). All centrality measures were computed on the thresholded network retaining only positive phi coefficient edges.

### 3.4 Additive interactions between condition pairs

Of the 66 condition pairs, 47 had at least 20 participants with both conditions and were tested; the 19 sparse pairs were suppressed as prespecified. The full set of joint counts, interaction contrasts, 95% confidence intervals and false-discovery-rate-adjusted *q*-values is given in Supplementary Table S8, and Supplementary Figure S1 displays the interaction heatmap with suppressed pairs masked. After Benjamini–Hochberg correction, one pair showed a greater-than-additive association with poor self-rated health: stroke combined with anemia (interaction contrast +0.55 on the risk-difference scale, 95% CI 0.24–0.86; *q* = 0.027; 22 participants with both conditions). No other pair remained statistically supported after correction (all remaining *q* ≥ 0.62), and estimates for rare combinations were imprecise. The interaction analysis should therefore be regarded as exploratory, with the stroke-anemia interaction its single robust finding.

### 3.5 Association between chronic conditions and poor self-rated health

In survey-weighted models adjusted for age, sex and residence (Table 3), the conditions most strongly associated with poor self-rated health were stroke (OR 6.51, 95% CI 2.75–15.44), heart attack (OR 3.14, 95% CI 1.55–6.36), tuberculosis (OR 3.10, 95% CI 1.63–5.91), asthma (OR 2.61, 95% CI 1.47–4.62), high blood cholesterol (OR 2.54, 95% CI 1.18–5.47), chronic pain (OR 2.37, 95% CI 1.71–3.28) and HIV (OR 2.16, 95% CI 1.63–2.85); anemia showed a weaker association (OR 1.47, 95% CI 1.03–2.11). Hypertension (OR 0.86, 95% CI 0.65–1.15), diabetes (OR 1.10, 95% CI 0.79–1.54), chronic bronchitis (OR 2.34, 95% CI 0.87–6.31) and cancer (OR 1.03, 95% CI 0.36–2.98) were not significantly associated with poor self-rated health after adjustment.

**Table 3:** Unadjusted and adjusted survey-weighted associations between chronic conditions, multimorbidity measures and poor self-rated health.

Predictor	Unadjusted OR (95% CI)	Adjusted OR (95% CI)
Tuberculosis	3.83 (1.97–7.42)	3.10 (1.63–5.91)
Hypertension	1.36 (1.04–1.76)	0.86 (0.65–1.15)
Stroke	9.63 (4.47–20.77)	6.51 (2.75–15.44)
Heart attack	4.79 (2.61–8.77)	3.14 (1.55–6.36)
Anemia	1.45 (1.06–1.97)	1.47 (1.03–2.11)
High blood cholesterol	3.83 (1.71–8.58)	2.54 (1.18–5.47)
Diabetes	1.73 (1.25–2.40)	1.10 (0.79–1.54)
Chronic bronchitis	4.37 (1.63–11.69)	2.34 (0.87–6.31)
Asthma	2.91 (1.59–5.33)	2.61 (1.47–4.62)
Cancer	1.56 (0.56–4.31)	1.03 (0.36–2.98)
HIV positive	2.38 (1.82–3.13)	2.16 (1.63–2.85)
Chronic pain	2.80 (2.05–3.84)	2.37 (1.71–3.28)
Disease count, per additional condition	1.70 (1.51–1.90)	1.50 (1.32–1.71)
Disease count, per 1 SD	1.94 (1.68–2.24)	1.66 (1.41–1.95)
Network score, per 1 SD	1.87 (1.62–2.15)	1.55 (1.31–1.82)
Max centrality, per 1 SD	1.48 (1.25–1.75)	1.24 (1.03–1.49)

Survey-weighted logistic regression incorporating the SADHS sampling weights, strata and primary sampling units (Taylor linearisation); *n* = 5,650. Adjusted models include age, sex and urban vs. rural residence. Continuous predictors are standardized (SD: disease count 1.25, network score 0.36, max centrality 0.37) and their odds ratios are per 1-SD increase. CI, confidence interval; OR, odds ratio; SD, standard deviation.

On a common per-1-SD scale, adjusted odds ratios were 1.66 (95% CI 1.41–1.95) for disease count (equivalently 1.50, 95% CI 1.32–1.71, per additional condition), 1.55 (95% CI 1.31–1.82) for the network score and 1.24 (95% CI 1.03–1.49) for max centrality. The network score was highly correlated with disease count (*r* = 0.96; variance inflation factors 13.6 and 14.5 in a joint model), and when disease count and both network measures were entered simultaneously the network-score coefficient inverted (OR 0.63 per SD, 95% CI 0.40–0.99), a pattern characteristic of collinearity; coefficients from the joint model are therefore not separately interpretable.

### 3.6 Model comparison

Discrimination and fit for the prespecified nested models are shown in Table 4. Adding disease count to the covariates increased the AUC from 0.721 (95% CI 0.702–0.740) to 0.758 (95% CI 0.740–0.777). The network score discriminated slightly but significantly less well than disease count (AUC 0.752 vs. 0.758; DeLong *p* < 0.001), as did max centrality (AUC 0.729; *p* < 0.001), and adding both network measures to disease count did not improve discrimination (AUC 0.756; DeLong *p* = 0.26), with a comparable design-based Akaike information criterion (2,783 vs. 2,789). The best-discriminating specification entered the 12 conditions individually (AUC 0.769, 95% CI 0.751–0.788; DeLong *p* = 0.001 vs. disease count). Ten-fold cross-validation, in which the network, centralities and individual-level scores were recomputed within each training fold, confirmed this ordering (mean held-out AUC: covariates only 0.720; disease count 0.762; network score 0.758; max centrality 0.738; disease count plus network measures 0.762; individual conditions 0.777; Supplementary Table S9). The network-derived individual measures therefore provided no incremental value beyond the disease count. Associations were consistent across alternative codings of the self-rated health outcome (Supplementary Table S10).

**Table 4:** Comparison of prespecified nested models predicting poor self-rated health.

Model	AUC (95% CI)	Design-based AIC	DeLong *p* vs. disease count	Cross-validated AUC, mean (SD)
Covariates only	0.721 (0.702–0.740)	2,876	—	0.720 (0.028)
Covariates + disease count	0.758 (0.740–0.777)	2,789	Reference	0.762 (0.032)
Covariates + network score	0.752 (0.733–0.770)	2,816	< 0.001	0.758 (0.035)
Covariates + max centrality	0.729 (0.711–0.748)	2,867	< 0.001	0.738 (0.036)
Covariates + disease count + network measures	0.756 (0.738–0.775)	2,783	0.26	0.762 (0.033)
Covariates + 12 individual conditions	0.769 (0.751–0.788)	2,752	0.001	0.777 (0.025)

All models are survey-weighted and include age, sex and urban vs. rural residence; continuous predictors per 1 SD. AUC computed from model-predicted probabilities, with DeLong 95% confidence intervals; DeLong tests are paired comparisons against the disease-count model. AIC is the design-based Akaike information criterion. Cross-validated AUC: 10-fold stratified cross-validation with the disease network, centralities and individual-level network measures recomputed within each training fold. AIC, Akaike information criterion; AUC, area under the receiver operating characteristic curve; SD, standard deviation.

## 4 Discussion

### 4.1 Main findings

This study applied network analysis to map multimorbidity patterns and their health impacts in a nationally representative sample of South African adults. Three key findings emerged. First, chronic conditions clustered into distinct patterns that varied across population subgroups. In the overall population, we identified three disease clusters: an infectious and hematological cluster (tuberculosis, HIV, anemia), a cardiometabolic and pain cluster (hypertension, diabetes, chronic pain), and a cardiovascular-respiratory-neoplastic cluster (stroke, heart attack, high cholesterol, chronic bronchitis, asthma, cancer). Notably, the infectious disease cluster remained stable across sex and residence subgroups. This is compatible with shared clinical or biological pathways linking HIV, tuberculosis and anemia that appear robust across the examined subgroups (17). However, other disease patterns showed marked heterogeneity. Males exhibited distinct separation of conditions by disease category, whereas females showed integration of cardiovascular events with metabolic conditions and chronic pain. Similarly, urban residents showed broad integration of metabolic, cardiac, and respiratory diseases, while rural residents showed respiratory conditions clustering with acute severe events rather than metabolic diseases. These subgroup differences have not been extensively documented in previous sub-Saharan African multimorbidity research. Label-permutation tests supported genuine subgroup differences in overall network structure (sex, *p* = 0.001; residence, *p* = 0.031).

Second, we identified specific conditions that occupy central positions within the multimorbidity network. Heart attack, chronic bronchitis, asthma, and chronic pain each co-occurred with 10 of the 11 other conditions examined (degree centrality 0.91) and were the most connected conditions in the network. Anemia had the highest betweenness centrality and therefore bridged the infectious cluster and the rest of the network, consistent with its multiple etiologies across infectious, nutritional, and chronic disease pathways (18). Whether high-centrality conditions could serve as practical markers of complex multimorbidity is a hypothesis requiring prospective evaluation rather than a conclusion these cross-sectional data can establish. Cross-sectional data cannot show whether high-centrality conditions precede or follow the accumulation of other diseases, and longitudinal cohorts would be needed to distinguish these (18).

Third, and contrary to our prespecified hypothesis, the network-based individual measures did not outperform the simple disease count. Once the survey design, covariate adjustment and a common per-1-SD scale were applied, the adjusted association for the network score (OR 1.55) was no stronger than that for disease count (OR 1.66); the two measures were correlated at *r* = 0.96; adding the network measures to disease count left discrimination unchanged (AUC 0.756 vs. 0.758); and cross-validation in which the network was re-derived within each training fold confirmed the absence of incremental value. This is an informative negative finding. Because the network score is a sum of centrality values across the conditions an individual has, it is mechanically entangled with the disease count, and its apparent superiority in unadjusted, unstandardised comparisons was an artifact of predictor scaling. In this population, the value of the network approach lay in describing the structure of multimorbidity—stable clusters, bridging conditions and subgroup differences—rather than in improving individual risk prediction beyond simple enumeration (8, 19).

More generally, our results illustrate the distinction between association, discrimination and prediction (20). Stroke carried the largest adjusted odds ratio yet contributes little to overall discrimination because it is rare (0.8% weighted prevalence); conversely, the modest per-SD odds ratio of the disease count belied its comparatively strong discrimination. A large odds ratio, particularly for a rare exposure or an unstandardised continuous score, is not evidence of predictive usefulness; model comparison should rest on discrimination, calibration and validated incremental performance rather than on coefficient magnitude.

### 4.2 Comparison with previous research

Our findings align with multimorbidity research identifying cardiovascular-metabolic clustering (21, 22), though the stable infectious cluster reflects South Africa's epidemiological profile (23). Our high-centrality conditions differ from high-income countries where mental health and musculoskeletal diseases dominate (24), likely reflecting prevalence differences and healthcare access patterns. In contrast to studies reporting superiority of network measures over disease counts (6, 25), we found no incremental predictive value of network measures once predictor scale and validation were addressed; and of the pairwise interactions we examined, only stroke combined with anemia was robust to false-discovery correction, differing from reports emphasizing respiratory or mental-physical interactions (26, 27).

### 4.3 Clinical and policy implications

Our findings do not support replacing simple disease counts with network-based individual risk scores: counting conditions remains a pragmatic and comparably informative marker of risk for poor self-rated health. The clustering results may nonetheless inform how services are organized. Conditions that reliably cluster together—hypertension, diabetes and chronic pain, and tuberculosis, HIV and anemia—are natural candidates for integrated management and co-screening pathways, an approach consistent with existing multimorbidity care models (28, 29) that requires prospective evaluation before implementation.

The sex-specific clustering patterns we observed suggest that multimorbidity assessment and management may need tailoring by sex. In females, the integration of heart attack with metabolic conditions and chronic pain suggests that cardiovascular risk assessment may need to consider pain and metabolic disease burden rather than treating these as separate clinical domains. In males, the distinct separation of disease categories suggests that screening protocols could be more modular, though integrated care remains essential when patients develop conditions across multiple clusters (27). These sex differences warrant validation in clinical cohorts before implementation but suggest that one-size-fits-all multimorbidity guidelines may miss important subgroup heterogeneity.

Health systems should prioritize integrated care for identified clusters. The cardiometabolic-pain cluster requires coordinated primary care protocols (30), while the infectious cluster demands continued HIV-TB-anemia service integration (31, 32). Urban-rural differences suggest geographic tailoring of service configurations.

Policy reforms should shift from vertical, single-disease programmes (33) toward horizontal integration (34). Addressing multimorbidity inequities requires upstream action on social determinants, including tobacco control, food environments and urban planning (35, 36).

### 4.4 Strengths and limitations

The SADHS uses a national probability sample, and hypertension, diabetes, anemia and HIV were confirmed by biomarker rather than self-report, which reduces underdiagnosis (37). The twelve-condition panel spans both the infectious and non-communicable burdens, and network methods have rarely been applied to multimorbidity in sub-Saharan Africa.

The cross-sectional design precludes causal inferences about disease trajectories (38). Absence of mental health data is a major limitation, likely underestimating multimorbidity complexity (39, 40). Self-reported health, while patient-centered (41), may be influenced by psychological and cultural factors. Self-reported conditions (tuberculosis, chronic bronchitis, asthma, cancer, chronic pain, stroke, heart attack) may underestimate prevalence (42), though biomarker confirmation for highly prevalent conditions mitigates this concern. Unmeasured confounding from health behaviors, socioeconomic status, and healthcare access remains possible. Because the adult health module was administered to one randomly selected adult per household, all prevalence and regression estimates apply the sampling weights, strata and primary sampling units; weighted and unweighted figures differ materially (for example, poor self-rated health 7.7% weighted vs. 13.2% unweighted), and the weighted values should be used for population-level statements. The analytic sample also required complete ascertainment of all 12 conditions, excluding 3,818 adults – principally those outside the biomarker subsamples – so complete-case selection may further limit representativeness. Interaction estimates for rare condition pairs rest on small numbers; we therefore suppressed the 19 pairs with fewer than 20 joint cases and applied false-discovery-rate correction across the 47 tested pairs, after which a single interaction remained supported. Self-rated health is a single-item global measure and is not equivalent to health-related quality of life as captured by multidimensional instruments. Finally, the network measures were derived from the same sample in which they were evaluated; we addressed this with cross-validation in which the network was recomputed within each training fold, and the conclusion – no incremental value over the disease count – was unchanged. The analytic rules for network construction (positive-phi edges, the minimum joint-count rule) were operational choices rather than prespecified criteria, although sensitivity analyses suggest the descriptive findings are robust to them. External validation in an independent population remains desirable.

## 5 Conclusions

In South African adults, chronic conditions form robust clusters – most notably a tuberculosis-HIV-anemia cluster that persisted across every subgroup and resampling analysis – and the structure of disease co-occurrence differs by sex and place of residence. Individual-level network measures, however, did not outperform a simple disease count as correlates of poor self-rated health once survey design, adjustment and validation were applied. Health systems in settings with dual infectious and non-communicable disease burdens may benefit from integrated care organized around reliably co-occurring conditions, while disease counts remain a pragmatic tool for identifying adults at risk of poor self-rated health; pattern-based measures merit prospective evaluation before clinical adoption.

## Data Availability

Dataset analyzed in this study is publicly available from the DHS Program following free registration at https://dhsprogram.com/data/Access-Instructions.cfm, matching the statement in the manuscript. Readers are therefore directed to the authoritative source rather than to the corresponding author.
