# Supplementary material for: Beyond disease counts: multimorbidity patterns and self-rated health in South African adults–a network analysis of the 2016 demographic and health survey
Source: Front Public Health. 2026 Oct 20;14:1947329. doi: 10.3389/fpubh.2026.1947329 (PMC13727725; doi:10.3389/fpubh.2026.1947329)
Supplement: Supplementary file 1 [file Table_1.docx]

**Supplementary Material**

Beyond Disease Counts: Multimorbidity Patterns and Self-Rated Health in South African Adults – A Network Analysis of the 2016 Demographic and Health Survey

*Contents: Supplementary Tables S1–S10, Supplementary Figure S1, STROBE checklist.*

# Supplementary Table S1. Operational definitions of the 12 chronic conditions

| **Condition** | **Ascertainment** | **Operational definition** |
| --- | --- | --- |
| Tuberculosis | Self-report | Ever told by a doctor, nurse or health worker that they had tuberculosis |
| Hypertension | Biomarker and/or treatment | Measured blood pressure ≥140/90 mmHg (average of repeated measurements per SADHS protocol) or current antihypertensive treatment |
| Stroke | Self-report | Ever told by a health professional that they had a stroke |
| Heart attack | Self-report | Ever told by a health professional that they had a heart attack |
| Anaemia | Biomarker | Haemoglobin <12 g/dL (women) or <13 g/dL (men), DHS altitude-adjusted values |
| High blood cholesterol | Self-report | Ever told by a health professional that they had high blood cholesterol |
| Diabetes | Biomarker and/or treatment | Glycated haemoglobin (HbA1c) ≥6.5% or current glucose-lowering treatment |
| Chronic bronchitis | Self-report | Ever told by a health professional that they had chronic bronchitis |
| Asthma | Self-report | Ever told by a health professional that they had asthma |
| Cancer | Self-report | Ever told by a health professional that they had cancer |
| HIV | Biomarker | Laboratory-confirmed positive HIV serostatus (SADHS HIV testing algorithm) |
| Chronic pain | Self-report | Persistent pain lasting more than 3 months |

*Self-reported conditions were collected in the SADHS adult health questionnaire; biomarker measurements followed the SADHS 2016 biomarker protocol. Exact variable derivations are available in the analysis code.*

# Supplementary Table S2. Participant flow and missing data

| **Step** | **n** |  |
| --- | --- | --- |
| Adult health module respondents | 10,336 |  |
| Excluded: aged under 18 years | 868 |  |
| Adults aged ≥18 years | 9,468 |  |
| Excluded: ≥1 of the 12 conditions not ascertainable | 3,818 |  |
| Analytic sample (complete-case) | 5,650 |  |

Missing data among the 9,468 adults, by variable (all other study variables, including self-rated health, age, sex and residence, were complete):

| **Variable** | **Missing, n** | **Missing, %** |
| --- | --- | --- |
| HIV serostatus | 3,485 | 36.8% |
| Diabetes (HbA1c) | 3,303 | 34.9% |
| Anaemia (haemoglobin) | 2,880 | 30.4% |
| Hypertension (blood pressure) | 2,187 | 23.1% |
| All self-reported conditions | 0 | 0.0% |

*Missingness reflects the SADHS biomarker subsample design. Participants could be missing more than one biomarker, so counts are not mutually exclusive.*

# Supplementary Table S3. Condition pairs with non-positive phi coefficients (no edge drawn)

| **Condition 1** | **Condition 2** | **Phi coefficient** |
| --- | --- | --- |
| Tuberculosis | Stroke | -0.0158 |
| Tuberculosis | Diabetes | -0.0025 |
| Tuberculosis | Cancer | -0.0134 |
| Hypertension | Anaemia | -0.0213 |
| Hypertension | HIV | -0.0434 |
| Stroke | HIV | -0.0014 |
| Heart attack | HIV | -0.0195 |
| Anaemia | High cholesterol | -0.0185 |
| High cholesterol | HIV | -0.0585 |
| Diabetes | HIV | -0.0590 |
| Chronic bronchitis | HIV | -0.0087 |
| Asthma | HIV | -0.0279 |
| Cancer | HIV | -0.0111 |
| HIV | Chronic pain | -0.0236 |

*The network retained all 52 pairs with phi > 0 as edges; the 14 pairs above (predominantly involving HIV) carried no edge.*

# Supplementary Table S4. Bootstrap co-clustering proportions (1,000 design-consistent replicates)

|  | **TB** | **HTN** | **STR** | **MI** | **ANA** | **CHOL** | **DM** | **CB** | **AST** | **CAN** | **HIV** | **PAIN** |
| --- | --- | --- | --- | --- | --- | --- | --- | --- | --- | --- | --- | --- |
| TB | – | 0.00 | 0.00 | 0.00 | 1.00 | 0.00 | 0.00 | 0.00 | 0.00 | 0.02 | 1.00 | 0.00 |
| HTN | 0.00 | – | 0.40 | 0.50 | 0.00 | 0.61 | 1.00 | 0.45 | 0.45 | 0.40 | 0.00 | 0.83 |
| STR | 0.00 | 0.40 | – | 0.84 | 0.00 | 0.77 | 0.40 | 0.88 | 0.88 | 0.97 | 0.00 | 0.52 |
| MI | 0.00 | 0.50 | 0.84 | – | 0.00 | 0.82 | 0.50 | 0.94 | 0.94 | 0.83 | 0.00 | 0.67 |
| ANA | 1.00 | 0.00 | 0.00 | 0.00 | – | 0.00 | 0.00 | 0.00 | 0.00 | 0.02 | 1.00 | 0.00 |
| CHOL | 0.00 | 0.61 | 0.77 | 0.82 | 0.00 | – | 0.61 | 0.84 | 0.83 | 0.75 | 0.00 | 0.63 |
| DM | 0.00 | 1.00 | 0.40 | 0.50 | 0.00 | 0.61 | – | 0.45 | 0.45 | 0.40 | 0.00 | 0.83 |
| CB | 0.00 | 0.45 | 0.88 | 0.94 | 0.00 | 0.84 | 0.45 | – | 0.98 | 0.85 | 0.00 | 0.61 |
| AST | 0.00 | 0.45 | 0.88 | 0.94 | 0.00 | 0.83 | 0.45 | 0.98 | – | 0.86 | 0.00 | 0.61 |
| CAN | 0.02 | 0.40 | 0.97 | 0.83 | 0.02 | 0.75 | 0.40 | 0.85 | 0.86 | – | 0.02 | 0.50 |
| HIV | 1.00 | 0.00 | 0.00 | 0.00 | 1.00 | 0.00 | 0.00 | 0.00 | 0.00 | 0.02 | – | 0.00 |
| PAIN | 0.00 | 0.83 | 0.52 | 0.67 | 0.00 | 0.63 | 0.83 | 0.61 | 0.61 | 0.50 | 0.00 | – |

*Proportion of 1,000 bootstrap replicates (primary sampling units resampled with replacement within strata) in which each pair of conditions was assigned to the same Louvain community. Pairs in the same main-analysis cluster co-clustered in a mean of 89% of replicates (minimum 75%) versus 21% for cross-cluster pairs. TB, tuberculosis; HTN, hypertension; STR, stroke; MI, heart attack; ANA, anaemia; CHOL, high cholesterol; DM, diabetes; CB, chronic bronchitis; AST, asthma; CAN, cancer; PAIN, chronic pain.*

# Supplementary Table S5. Sensitivity analysis: observed-to-expected co-occurrence network

| **Quantity** | **Value** |
| --- | --- |
| Edge rule | O/E > 1, weight = log(O/E) |
| Edges retained | 52 of 66 |
| Spearman correlation of edge weights with phi network | 0.80 |
| Spearman correlation of eigenvector centrality ranking with main analysis | 1.00 |
| Adjusted Rand index of community partition vs main analysis | 0.49 |
| Infectious cluster (TB–HIV–anaemia) | Identical to main analysis |
| Non-communicable clusters | Merged into a single community under O/E weighting |

# Supplementary Table S6. Sensitivity analysis: survey-weighted phi network

| **Quantity** | **Value** |
| --- | --- |
| Edges retained (weighted phi > 0) | 56 of 66 |
| Pearson correlation of edge weights with unweighted network | 0.83 |
| Spearman correlation of eigenvector centrality ranking with main analysis | 0.83 |
| Adjusted Rand index of community partition vs main analysis | 0.28 |
| Infectious cluster (TB–HIV–anaemia) | Identical to main analysis |
| Non-communicable clusters | Heart attack grouped with respiratory-pain conditions; hypertension, diabetes, cholesterol, stroke and cancer formed the second cluster |

# Supplementary Table S7. Permutation tests for subgroup network differences

| **Comparison** | **Observed summed \|edge-weight difference\|** | **Permutations** | **p-value** |
| --- | --- | --- | --- |
| Male vs female | 2.67 | 1,000 | 0.001 |
| Urban vs rural | 2.19 | 1,000 | 0.031 |

*Group labels were permuted 1,000 times; both subgroup networks were re-estimated at each iteration and the summed absolute difference in phi coefficients across all 66 pairs was compared with the permutation distribution.*

# Supplementary Table S8. Additive interactions for all 66 condition pairs

| **Condition 1** | **Condition 2** | **Joint n** | **Interaction contrast** | **95% CI** | **p** | **q (BH-FDR)** |
| --- | --- | --- | --- | --- | --- | --- |
| Anaemia | Asthma | 47 | -0.143 | (-0.306 to 0.019) | 0.083 | 0.654 |
| Anaemia | Cancer | 20 | 0.010 | (-0.226 to 0.246) | 0.935 | 0.986 |
| Anaemia | Chronic bronchitis | 17 | Suppressed | – | – | – |
| Anaemia | Chronic pain | 295 | 0.005 | (-0.080 to 0.090) | 0.903 | 0.986 |
| Anaemia | Diabetes | 313 | 0.008 | (-0.073 to 0.089) | 0.847 | 0.986 |
| Anaemia | High cholesterol | 31 | 0.195 | (-0.207 to 0.597) | 0.340 | 0.826 |
| Anaemia | HIV | 386 | 0.072 | (0.009 to 0.136) | 0.025 | 0.595 |
| Asthma | Cancer | 7 | Suppressed | – | – | – |
| Asthma | Chronic pain | 83 | -0.005 | (-0.210 to 0.200) | 0.961 | 0.986 |
| Asthma | HIV | 33 | -0.003 | (-0.216 to 0.210) | 0.978 | 0.986 |
| Cancer | Chronic pain | 20 | -0.003 | (-0.342 to 0.336) | 0.986 | 0.986 |
| Cancer | HIV | 10 | Suppressed | – | – | – |
| Chronic bronchitis | Asthma | 21 | -0.013 | (-0.418 to 0.391) | 0.949 | 0.986 |
| Chronic bronchitis | Cancer | 5 | Suppressed | – | – | – |
| Chronic bronchitis | Chronic pain | 38 | 0.128 | (-0.223 to 0.480) | 0.474 | 0.856 |
| Chronic bronchitis | HIV | 14 | Suppressed | – | – | – |
| Diabetes | Asthma | 82 | 0.165 | (-0.088 to 0.418) | 0.201 | 0.826 |
| Diabetes | Cancer | 24 | -0.120 | (-0.311 to 0.072) | 0.222 | 0.826 |
| Diabetes | Chronic bronchitis | 31 | 0.047 | (-0.364 to 0.457) | 0.823 | 0.986 |
| Diabetes | Chronic pain | 387 | 0.019 | (-0.071 to 0.108) | 0.684 | 0.946 |
| Diabetes | HIV | 219 | -0.020 | (-0.094 to 0.055) | 0.605 | 0.917 |
| Heart attack | Anaemia | 61 | 0.071 | (-0.217 to 0.359) | 0.628 | 0.922 |
| Heart attack | Asthma | 41 | -0.111 | (-0.401 to 0.179) | 0.452 | 0.851 |
| Heart attack | Cancer | 12 | Suppressed | – | – | – |
| Heart attack | Chronic bronchitis | 23 | 0.269 | (-0.104 to 0.642) | 0.157 | 0.826 |
| Heart attack | Chronic pain | 128 | 0.020 | (-0.229 to 0.270) | 0.873 | 0.986 |
| Heart attack | Diabetes | 113 | 0.109 | (-0.157 to 0.375) | 0.422 | 0.826 |
| Heart attack | High cholesterol | 36 | 0.183 | (-0.214 to 0.580) | 0.366 | 0.826 |
| Heart attack | HIV | 42 | 0.151 | (-0.150 to 0.452) | 0.326 | 0.826 |
| High cholesterol | Asthma | 20 | 0.151 | (-0.360 to 0.662) | 0.562 | 0.908 |
| High cholesterol | Cancer | 6 | Suppressed | – | – | – |
| High cholesterol | Chronic bronchitis | 23 | 0.125 | (-0.413 to 0.663) | 0.649 | 0.924 |
| High cholesterol | Chronic pain | 73 | 0.150 | (-0.193 to 0.493) | 0.390 | 0.826 |
| High cholesterol | Diabetes | 91 | 0.237 | (-0.016 to 0.490) | 0.066 | 0.619 |
| High cholesterol | HIV | 14 | Suppressed | – | – | – |
| HIV | Chronic pain | 234 | 0.050 | (-0.045 to 0.144) | 0.302 | 0.826 |
| Hypertension | Anaemia | 552 | 0.010 | (-0.047 to 0.067) | 0.735 | 0.960 |
| Hypertension | Asthma | 129 | 0.198 | (-0.009 to 0.405) | 0.061 | 0.619 |
| Hypertension | Cancer | 39 | 0.037 | (-0.178 to 0.253) | 0.734 | 0.960 |
| Hypertension | Chronic bronchitis | 55 | 0.149 | (-0.196 to 0.495) | 0.396 | 0.826 |
| Hypertension | Chronic pain | 720 | 0.056 | (-0.015 to 0.127) | 0.125 | 0.826 |
| Hypertension | Diabetes | 899 | 0.021 | (-0.043 to 0.084) | 0.525 | 0.908 |
| Hypertension | Heart attack | 184 | 0.053 | (-0.135 to 0.242) | 0.579 | 0.908 |
| Hypertension | High cholesterol | 156 | 0.135 | (-0.106 to 0.375) | 0.272 | 0.826 |
| Hypertension | HIV | 514 | -0.034 | (-0.090 to 0.022) | 0.231 | 0.826 |
| Hypertension | Stroke | 67 | -0.047 | (-0.416 to 0.322) | 0.803 | 0.986 |
| Stroke | Anaemia | 22 | 0.548 | (0.237 to 0.858) | <0.001 | 0.027 |
| Stroke | Asthma | 16 | Suppressed | – | – | – |
| Stroke | Cancer | 8 | Suppressed | – | – | – |
| Stroke | Chronic bronchitis | 5 | Suppressed | – | – | – |
| Stroke | Chronic pain | 35 | 0.238 | (-0.121 to 0.597) | 0.193 | 0.826 |
| Stroke | Diabetes | 32 | -0.042 | (-0.483 to 0.400) | 0.853 | 0.986 |
| Stroke | Heart attack | 22 | -0.302 | (-0.744 to 0.140) | 0.180 | 0.826 |
| Stroke | High cholesterol | 17 | Suppressed | – | – | – |
| Stroke | HIV | 17 | Suppressed | – | – | – |
| Tuberculosis | Anaemia | 25 | -0.072 | (-0.305 to 0.162) | 0.546 | 0.908 |
| Tuberculosis | Asthma | 8 | Suppressed | – | – | – |
| Tuberculosis | Cancer | 0 | Suppressed | – | – | – |
| Tuberculosis | Chronic bronchitis | 3 | Suppressed | – | – | – |
| Tuberculosis | Chronic pain | 30 | 0.291 | (0.013 to 0.568) | 0.040 | 0.619 |
| Tuberculosis | Diabetes | 21 | 0.199 | (-0.208 to 0.606) | 0.338 | 0.826 |
| Tuberculosis | Heart attack | 12 | Suppressed | – | – | – |
| Tuberculosis | High cholesterol | 3 | Suppressed | – | – | – |
| Tuberculosis | HIV | 54 | -0.111 | (-0.377 to 0.154) | 0.411 | 0.826 |
| Tuberculosis | Hypertension | 46 | 0.127 | (-0.143 to 0.397) | 0.355 | 0.826 |
| Tuberculosis | Stroke | 0 | Suppressed | – | – | – |

*Interaction contrasts on the risk-difference scale from survey-weighted linear-probability models. Pairs with fewer than 20 participants having both conditions were suppressed as prespecified (19 of 66). One pair remained supported after Benjamini–Hochberg correction: stroke × anaemia (q = 0.027).*

# Supplementary Table S9. Ten-fold cross-validated discrimination

| **Model** | **Mean held-out AUC** | **SD across folds** |
| --- | --- | --- |
| Covariates only | 0.720 | 0.028 |
| Covariates + disease count | 0.762 | 0.032 |
| Covariates + network score | 0.758 | 0.035 |
| Covariates + max centrality | 0.738 | 0.036 |
| Covariates + disease count + network measures | 0.762 | 0.033 |
| Covariates + 12 individual conditions | 0.777 | 0.025 |

*Stratified 10-fold cross-validation; within each training fold the phi network, centralities, and individual-level network score and max centrality were recomputed before evaluation on the held-out fold, so no network information leaks from test to training data.*

# Supplementary Table S10. Sensitivity of associations to the outcome coding

| **Predictor** | **Poor vs average/good/excellent (primary)** | **Poor/average vs good/excellent** | **Poor/average/good vs excellent** |
| --- | --- | --- | --- |
| Disease count (per 1 SD) | 1.66 (1.41–1.95) | 1.55 (1.39–1.73) | 1.32 (1.12–1.55) |
| Network score (per 1 SD) | 1.55 (1.31–1.82) | 1.52 (1.36–1.69) | 1.27 (1.08–1.51) |
| Max centrality (per 1 SD) | 1.24 (1.03–1.49) | 1.22 (1.12–1.32) | 1.13 (1.02–1.25) |

*Survey-weighted odds ratios (95% CI) adjusted for age, sex and residence, at each cumulative cut of the four-category self-rated health scale. The consistent ordering across cuts indicates the proportional-odds pattern approximately holds and that conclusions are robust to the dichotomisation.*

# Supplementary Figure S1. Additive interactions between condition pairs

*Heatmap of interaction contrasts (risk-difference scale) for poor self-rated health. Grey hatched cells are suppressed pairs (fewer than 20 participants with both conditions; 19 of 66 pairs). The black outline marks the single pair statistically supported after Benjamini–Hochberg false-discovery-rate correction (stroke × anaemia, interaction contrast +0.55, 95% CI 0.24–0.86, q = 0.027). Estimates from survey-weighted linear-probability models.*

# STROBE checklist (cross-sectional studies)

| **Item** | **Section** | **Recommendation (abbreviated)** | **Where addressed** |
| --- | --- | --- | --- |
| 1 | Title and abstract | Study design in title/abstract; informative abstract | Title page; Abstract |
| 2 | Background/rationale | Scientific background and rationale | Introduction, paragraphs 1–2 |
| 3 | Objectives | Specific objectives and prespecified hypotheses | Introduction, final paragraph |
| 4 | Study design | Key elements of study design | Methods, Study design and population |
| 5 | Setting | Setting, locations, relevant dates | Methods, Study design and population |
| 6 | Participants | Eligibility criteria, sources, selection | Methods, Study design and population (participant flow) |
| 7 | Variables | Outcomes, exposures, covariates defined | Methods, Outcome and exposures; Supplementary Table S1 |
| 8 | Data sources/measurement | Sources and methods of assessment | Methods, Outcome and exposures; Supplementary Table S1 |
| 9 | Bias | Efforts to address potential bias | Methods (validation, FDR); Discussion, Strengths and limitations |
| 10 | Study size | How study size was arrived at | Methods, Study design and population |
| 11 | Quantitative variables | Handling of quantitative variables | Methods, Statistical analysis (standardisation, coding) |
| 12 | Statistical methods | All methods incl. subgroups, missing data, sensitivity | Methods, Statistical analysis; Supplementary Tables S2, S5–S10 |
| 13 | Participants | Numbers at each stage; reasons for non-participation | Methods (participant flow); Supplementary Table S2 |
| 14 | Descriptive data | Characteristics, missing data per variable | Table 1; Results, Sample characteristics; Supplementary Table S2 |
| 15 | Outcome data | Numbers of outcome events | Table 1; Results |
| 16 | Main results | Unadjusted and adjusted estimates with CIs | Tables 3–4; Results |
| 17 | Other analyses | Subgroups, interactions, sensitivity analyses | Results; Supplementary Tables S4–S10, Figure S1 |
| 18 | Key results | Summary with reference to objectives | Discussion, Main findings |
| 19 | Limitations | Limitations, direction and magnitude of bias | Discussion, Strengths and limitations |
| 20 | Interpretation | Cautious overall interpretation | Discussion; Conclusions |
| 21 | Generalisability | External validity | Discussion, Strengths and limitations |
| 22 | Funding | Source of funding and role of funders | Statements, Funding |
